# Establishment and validation of a nomogram model for early diagnosis of gastric cancer: a large-scale cohort study

**DOI:** 10.3389/fonc.2024.1463480

**Published:** 2024-11-29

**Authors:** Haiyu Wang, Yumin Ding, Shujing Zhao, Kaixu Li, Dehong Li

**Affiliations:** ^1^ School of Public Health, Gansu University of Chinese Medicine, Lanzhou, Gansu, China; ^2^ Department of Clinical Laboratory, Gansu Provincial Hospital, Lanzhou, Gansu, China

**Keywords:** gastric cancer, nomogram model, early diagnosis, marker, characteristic pattern

## Abstract

**Purpose:**

Identifying high-risk populations and diagnosing gastric cancer (GC) early remains challenging. This study aimed to establish and verify a nomogram model for the early diagnosis of GC based on conventional laboratory indicators.

**Methods:**

We performed a retrospective analysis of the clinical data of 2,770 individuals with first diagnosis of GC and 1,513 patients with benign gastric disease from January 2018 to December 2022. The cases were divided into the training set and validation set randomly, with a ratio of 7:3. Variable screening was performed by least absolute shrinkage and selection operator (LASSO) and logistic regression analysis. A nomogram was constructed in the training set to assist in the early diagnosis of GC.

**Results:**

There were 4283 patients included in the study, with 2998 patients assigned in the training set and 1285 patients in the validation set. Through LASSO regression and logistic regression analysis, independent variables associated with GC were identified, including CEA, CA199, LYM, HGB, MCH, MCHC, PLT, ALB, TG, HDL, and AFR. The nomogram model was constructed using the above 11 independent indicators. The AUC was 0.803 for the training set and 0.797 for the validation set, indicating that the model showed high clinical diagnostic efficacy. The calibration curves and decision curve analysis (DCA) of the nomogram presented good calibration and clinical application ability.

**Conclusion:**

Based on the analysis of large sample size, we constructed a nomogram model with 11 routine laboratory indicators, which showed good discrimination ability and calibration.

## Introduction

Gastric cancer, one of the most common malignant tumors globally, ranks fourth among the causes of cancer-related deaths worldwide (1). According to statistics, approximately half of the GC deaths worldwide occur in China, imposing a heavy burden (1). Due to the lack of specific clinical symptoms and signs in the early stages, as well as the absence of effective biomarkers and screening methods, most GC patients are diagnosed in late or metastatic stages, with low resection rates and poor prognosis (2, 3). Therefore, it is crucial to conduct early screening, diagnosis, and treatment in order to improve the survival rate and prognosis of patients with GC.

Currently, endoscopic examination combined with histopathological evaluation of tissues is the gold standard for clinical diagnosis of GC (4). However, endoscopic examination is invasive and has a certain rate of missed diagnosis, and the tolerance and compliance of the population are relatively poor, limiting its routine use in GC screening (5, 6). Serum biomarker detection has the advantages of non-invasiveness, minimally invasive procedures, ease of operation, and convenient dynamic monitoring, making it easily accepted by subjects and widely used clinically (7). Currently, the sensitivity and specificity of single indicators cannot meet the demands (8), and the clinical diagnostic performance of multiple routine blood indicators combined detection remains unsatisfactory. In recent years, research on tumor diagnostic methods has shifted from searching for single biomarkers to finding a specific group of markers, also known as “characteristic patterns” (9, 10). The establishment of biomarker characteristic patterns for early diagnosis of GC can provide new ideas for research on tumor diagnostic methods.

Nomograms, as a reliable and convenient tool for quantifying significant risk factors, have been widely used in clinical practice (11, 12). Currently, there are few reports on the application of Nomogram models for GC diagnosis, with more studies focusing on prognosis and metastasis of GC (13, 14). Based on large-sample case data, this study constructs and validates an early GC diagnosis model, screening out a sensitive and specific group of early GC diagnosis biomarkers, aiming to provide effective references for clinical screening and early diagnosis of GC.

## Materials and methods

### Study subjects

**Figure 1:**
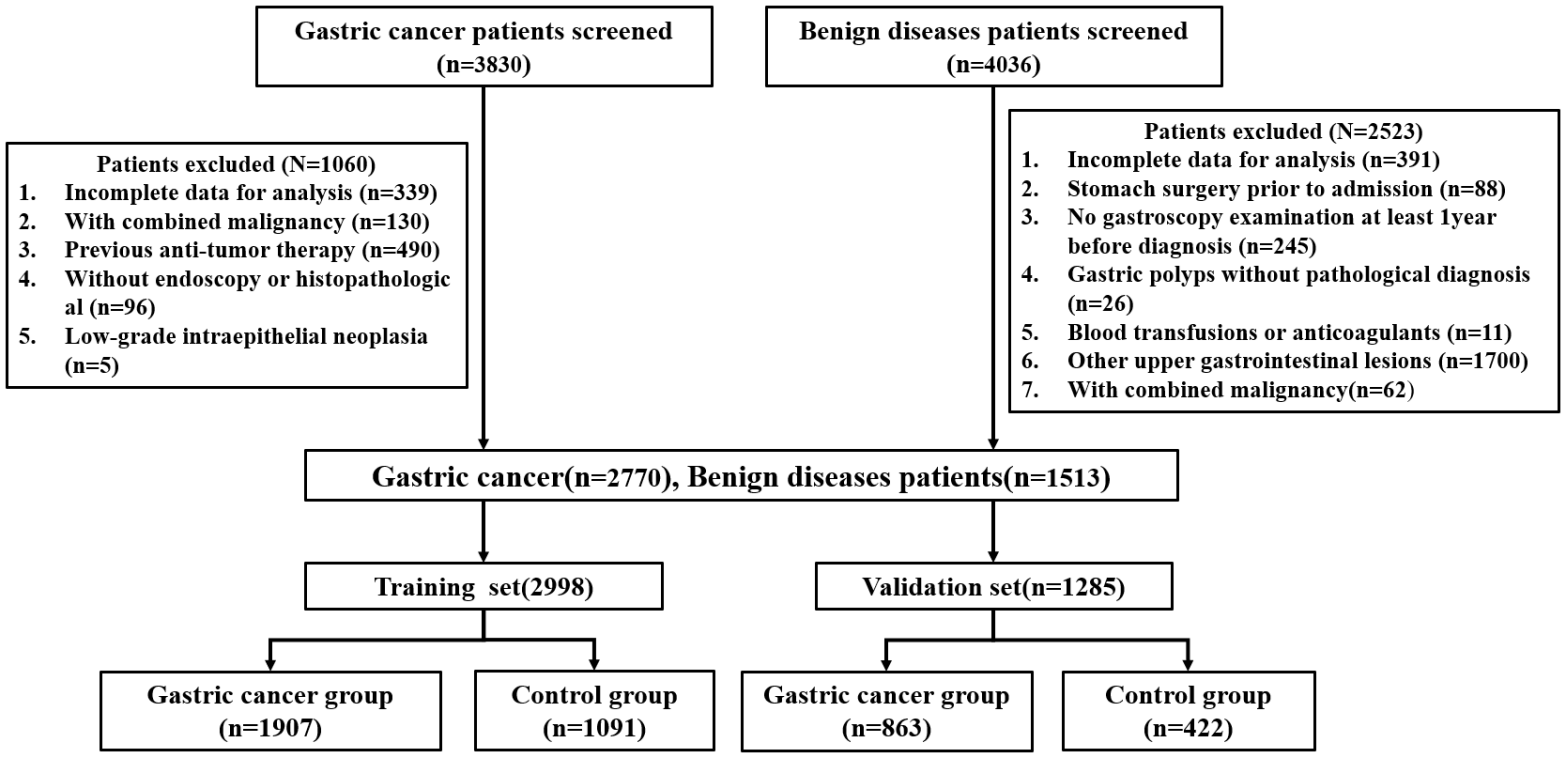
Flowchart of enrolled patients screening in the study.

This retrospective study collected the clinical data of patients (n=7,866) with gastrointestinal diseases who were admitted at Gansu Provincial Hospital from January 2018 to December 2022. The study flowchart is represented in **Figure 1**. There were 4,283 patients who met the inclusion criteria, including 2770 patients with GC and 1513 patients with benign gastric disease (gastritis, gastric ulcer, gastric polyp). At random, all patients were divided into a training set and a validation set at a 7:3 ratio. The study was approved by The Medical Ethics Committee of the Gansu provincial Hospital (2024–306).

According to the 2022 NCCN clinical practice guidelines for GC, histopathological biopsy is the gold standard for the diagnosis of all cases of GC (15). Gastritis, gastric ulcers, and gastric polyps were all diagnosed by gastroscopy, and biopsies were performed on polyps seen under the microscope. Inclusion criteria for the case group: ①Patients confirmed with GC by pathological report; ②No chemotherapy, radiotherapy, surgery, or other anti-tumor treatments before admission; ③No history of other malignant tumors before admission; ④Patients with complete clinical information and medical records. The exclusion criteria for the case and control groups are shown in **Figure 1**.

### Data collection

Collected data included demographic variables and laboratory findings of the study population. Demographic variables collected for the study included age and sex. The laboratory findings of all patients were obtained from their initial routine serological examination upon admission for treatment, including tumor markers and routine tests that could reflect the inflammation, abnormal metabolic and coagulation function of patients (16–19). The above indicators include the levels of tumor biomarkers [carcinoembryonic antigen (CEA), alpha-fetoprotein (AFP), carbohydrate antigen 19-9 (CA199), and carbohydrate antigen 125 (CA125)], infectivity index [white blood cells (WBC), lymphocytes (LYM), neutrophil cell (NEUT), monocyte(MO), eosinophilic cell (EOS), platelet-lymphocyte ratio (PLR), neutrophil-lymphocyte ratio (NLR), lymphocyte-monocyte ratio (LMR), red blood cell (RBC), hemoglobin (HGB), mean red blood cell volume (MCV), mean red blood cell hemoglobin content (MCH), mean red blood cell hemoglobin concentration (MCHC), red blood cell distribution width-SD (RDW-SD), and red blood cell distribution width-CV (RDW-CV)], coagulation index [prothrombin activity (PTA), prothrombin time (PT), activated partial thromboplastin time (APTT), international normalized ratio (INR), thrombin time (TT), platelet (PLT), fibrinogen (FIB), platelet volume distribution width (PDW), mean platelet volume (MPV), and albumin/fibrinogen (AFR)], lipid metabolism index [triglycerides (TG), cholesterol (TC), high density lipoprotein cholesterol (HDL-C), and low density lipoprotein cholesterol (LDL-C)], and nutritional index [albumin (ALB), globulin (GLB), and albumin/globulin (AGR)].

### Variable selection and data processing

Based on existing literatures and clinical expertise, we identified 46 potential predictor variables. In our study, 10 variables with missing values exceeding 20% were excluded, including PG I, PG II, PG I/II, CA724, NSE, D-dimer, creatine kinase (CK), lactate dehydrogenase (LDH), creatine kinase isoenzyme (CK-MB), and homocysteine (HCY). Considering that the missing data were missing at random and these variables are numerical variables, we adopted the prediction mean matching (PMM) from multiple interpolation technology to fill the 36 indicators with missing values less than 20%, so as to make the data as complete as possible and improve the prediction ability of the model.

### Statistical analysis

The SPSS 26.0 and R 4.3.2 software programs were used for statistical analyses and data processing. (1) The included 36 variables were all numerical variables, and predictive mean matching was used to impute a small amount of missing data. (2) Chi-square tests were employed to analyze the differences in distribution among groups for categorical variables; there were no quantitative variables that followed the normal distribution, which were described as median (interquartile range), and the group comparison was conducted using the Mann-Whitney U test. (3) Utilizing LASSO regression to screen for optimal parameters, aiming to minimize potential collinearity among measured variables from the same patient and avoid overfitting of variables. (4) Univariate and multivariate logistic regression analyses were employed to screen various indicator data and obtain meaningful risk factors for early GC diagnosis. (5) Based on the independent relevant factors determined by multivariate logistic regression analysis, a nomogram for early diagnosis of GC was developed using the ‘rms’ package in R. (6) The model’s discrimination, calibration, and clinical utility were validated and evaluated using ROC curve, AUC, calibration curve, and DCA.

## Results

### Clinical characteristics

According to the inclusion and exclusion criteria, a total of 4283 patients were enrolled in this study. All patients were randomly divided into the training set (n = 2998) and the validation set (n = 1285). The demographic and clinical data of the populations in the training and validation sets are shown in **Table 1**. There were no statistically significant differences in age, sex and 36 routine laboratory parameters in tumor markers, infectivity index, coagulation index, lipid metabolism index and nutritional index (P > 0.05), indicating the randomness and rationality of the grouping. In the training set, there were 1907 cases of GC patients in the case group and 1091 cases of benign gastric diseases in the control group. The statistical analysis results of general demographic data and routine laboratory tests between the case and control groups are shown in **Table 2**. There were statistically significant differences between the two groups in terms of age and sex, and patients with GC showed a higher age (median 62.0 years vs 57.0 years). In addition, the levels of AFP, EOS, PTA, INR, PT, APTT and LDL were not statistically significant between the two groups, while the levels of the other 29 conventional laboratory indicators were significantly different.

**Table 1:** Demographics and clinical indicators of study participants in the training and validation sets.

Variables	Train N=2998	Test N=1285	*χ^2^/Z*	*P*
Age	61.00 (52.00, 68.00)	60.00 (52.00, 68.00)	-0.102	0.919
Sex:			1.139	0.286
Male	2048(68.30%)	899 (70.00%)		
Female	950(31.70%)	386 (30.00%)		
AFP	2.37 (1.75, 3.43)	2.36 (1.71, 3.37)	-0.603	0.546
CEA	2.19 (1.37, 4.10)	2.32 (1.40, 4.39)	-1.371	0.170
CA125	12.60 (8.52, 21.55)	13.00 (8.70, 22.10)	-0.891	0.373
CA199	6.10 (2.64, 16.93)	6.44 (2.64, 19.87)	-0.976	0.329
WBC	5.70 (4.50, 7.20)	5.70 (4.60, 7.10)	-0.256	0.798
NEUT	3.62 (2.69, 4.90)	3.62 (2.73, 4.92)	-0.670	0.503
LYM	1.37 (1.01, 1.75)	1.36 (1.02, 1.74)	-0.588	0.556
MO	0.40 (0.31, 0.52)	0.41 (0.31, 0.53)	-1.026	0.305
EOS	0.06 (0.03, 0.12)	0.06 (0.03, 0.12)	-0.943	0.345
RBC	4.49 (3.87, 4.95)	4.43 (3.80, 4.95)	-0.935	0.350
HGB	136.00 (108.00, 152.00)	135.00 (108.00, 152.00)	-0.125	0.900
MCV	90.20 (85.88, 94.00)	90.30 (86.40, 93.90)	-0.813	0.416
MCH	30.40 (28.30, 31.90)	30.40 (28.60, 31.80)	-0.109	0.913
MCHC	333.00 (321.00, 344.00)	333.00 (321.00, 343.00)	-0.240	0.811
RDW-SD	43.90 (41.30, 47.70)	44.00 (41.20, 47.60)	-0.016	0.987
RDW-CV	13.30 (12.60, 14.90)	13.30 (12.60, 14.75)	-0.440	0.660
PLT	208.00 (160.00, 266.00)	208.00 (162.00, 264.00)	-0.110	0.913
MPV	11.00 (10.00, 12.00)	11.00 (10.00, 12.00)	-0.498	0.618
PDW	13.00 (11.00, 16.00)	13.00 (11.00, 16.00)	-0.011	0.991
PTA	97.00 (89.00, 106.00)	98.00 (89.50, 106.00)	-0.076	0.939
INR	1.01 (0.97, 1.07)	1.01 (0.97, 1.07)	-0.409	0.682
PT	13.30 (12.80, 13.90)	13.30 (12.70, 13.90)	-0.244	0.807
APTT	36.90 (34.20, 39.70)	36.90 (34.00,39.80)	-1.180	0.238
FIB	3.29 (2.74, 4.05)	3.33 (2.77, 4.11)	-1.084	0.278
TT	16.60 (15.70, 17.50)	16.70 (15.80, 17.50)	-1.518	0.129
ALB	39.70 (35.70, 42.90)	39.30 (35.40, 42.79)	-1.595	0.111
GLB	27.00 (23.90, 30.00)	27.00 (23.93, 30.30)	-0.064	0.949
AGR	1.47 (1.29, 1.67)	1.46 (1.27, 1.66)	-1.234	0.217
TC	3.92 (3.31, 4.58)	3.92 (2.89, 4.57)	-0.183	0.855
TG	1.14 (0.89, 1.56)	1.15 (0.89, 1.59)	-0.744	0.457
HDL	1.02 (0.86, 1.20)	1.01 (0.85, 1.20)	-0.654	0.513
LDL	2.26 (1.79, 2.77)	2.25 (1.79, 2.76)	-0.274	0.784
NLR	2.64 (1.80, 4.04)	2.68 (1.87, 4.13)	-1.461	0.144
PLR	149.47 (107.21, 218.14)	150.37 (110.63, 214.15)	-0.665	0.506
LMR	3.47 (2.48, 4.63)	3.38 (2.42, 4.57)	-1.815	0.070
AFR	11.96 (9.16, 15.02)	11.82 (8.86, 14.91)	-1.457	0.145

**Table 2:** Demographics and clinical indicators of the GC group and control groups in the training set.

Variables	GC N=1907	Control N=1091	*χ^2^/Z*	*P*
Age	62.00 (54.00, 69.00)	57.00 (49.00, 67.00)	-8.320	<0.001
Sex:			213.973	<0.001
Male	1482 (77.70%)	566 (51.90%)		
Female	425 (22.30%)	525 (48.10%)		
AFP	2.36 (1.72, 3.54)	2.38 (1.78, 3.27)	-0.508	0.612
CEA	2.68 (1.58, 6.64)	1.69 (1.15, 2.51)	-17.248	<0.001
CA125	14.20 (9.10, 28.10)	10.80 (7.80, 15.40)	-11.948	<0.001
CA199	7.40 (2.94, 34.86)	4.75 (2.26, 9.67)	-10.790	<0.001
WBC	5.80 (4.70, 7.10)	5.50 (4.40, 7.20)	-2.312	<0.001
NEUT	3.75 (2.82, 4.99)	2.38 (2.48, 4.70)	-5.027	<0.001
LYM	1.31 (0.98, 1.66)	1.46 (1.10, 1.89)	-6.939	<0.001
MO	0.41 (0.31, 0.53)	0.38 (0.29, 0.50)	-4.164	<0.001
EOS	0.06 (0.03, 0.11)	0.06 (0.03, 0.12)	-1.095	0.274
RBC	4.43 (3.79, 4.92)	4.56 (4.40, 5.01)	-3.512	<0.001
HGB	132.00 (102.00, 150.00)	141.00 (120.00, 155.00)	-7.035	<0.001
MCV	89.30 (84.00, 93.60)	91.10 (87.80, 94.60)	-8.639	<0.001
MCH	29.90 (27.00, 31.70)	31.00 (29.60, 32.20)	-11.625	<0.001
MCHC	331.00 (315.00, 342.00)	338.00 (328.00, 347.00)	-11.971	<0.001
RDW-SD	44.20 (41.60, 48.00)	43.50 (40.90, 47.00)	-4.561	<0.001
RDW-CV	13.50 (12.70, 15.50)	13.10 (12.40, 14.20)	-8.606	<0.001
PLT	217.00 (170.00, 279.00)	193.00 (148.00, 242.00)	-9.141	<0.001
MPV	11.00 (10.00, 12.00)	11.00 (10.00, 12.00)	-5.541	<0.001
PDW	13.00 (11.00, 15.00)	14.00 (12.00, 16.00)	-6.291	<0.001
PTA	97.00 (89.00, 105.00)	98.00 (89.00, 107.00)	-1.125	0.260
INR	1.02 (0.97, 1.07)	1.01 (0.96, 1.07)	-0.311	0.311
PT	13.30 (12.90, 13.90)	13.30 (12.70, 13.90)	-1.007	0.314
APTT	36.80 (34.30, 39.60)	36.90 (34.00,39.90)	-0.516	0.606
Fbg	3.52 (2.94, 4.29)	2.96 (2.47, 3.55)	-15.790	<0.001
TT	16.50 (15.60, 17.40)	16.80 (16.00, 17.70)	-5.818	<0.001
ALB	38.60 (34.70, 41.80)	41.60 (38.00, 44.30)	-14.614	<0.001
GLB	27.10 (24.10, 30.10)	26.71 (23.60, 29.87)	-2.762	<0.001
AGR	1.42 (1.24, 1.61)	1.56 (1.37, 1.75)	-12.167	<0.001
TC	3.82 (3.27, 4.42)	4.09 (3.40, 4.79)	-5.853	<0.001
TG	1.11 (0.88, 1.44)	1.25 (0.90, 1.77)	-6.190	<0.001
HDL	1.00 (0.85, 1.17)	1.06 (0.87, 1.26)	-5.497	<0.001
LDL	2.24 (1.79, 2.73)	2.32 (1.79, 2.82)	-1.676	0.094
NLR	2.82 (1.96, 4.28)	2.30 (1.60, 3.65)	-8.495	<0.001
PLR	164.91 (116.96, 239.19)	128.99 (93.02, 174.19)	-12.498	<0.001
LMR	3.23 (2.30, 4.37)	3.89 (2.88, 5.06)	-9.763	<0.001
AFR	10.85 (8.45, 13.46)	14.03 (11.26, 16.84)	-18.381	<0.001

### Univariate regression and LASSO regression analysis

**Figure 2:**
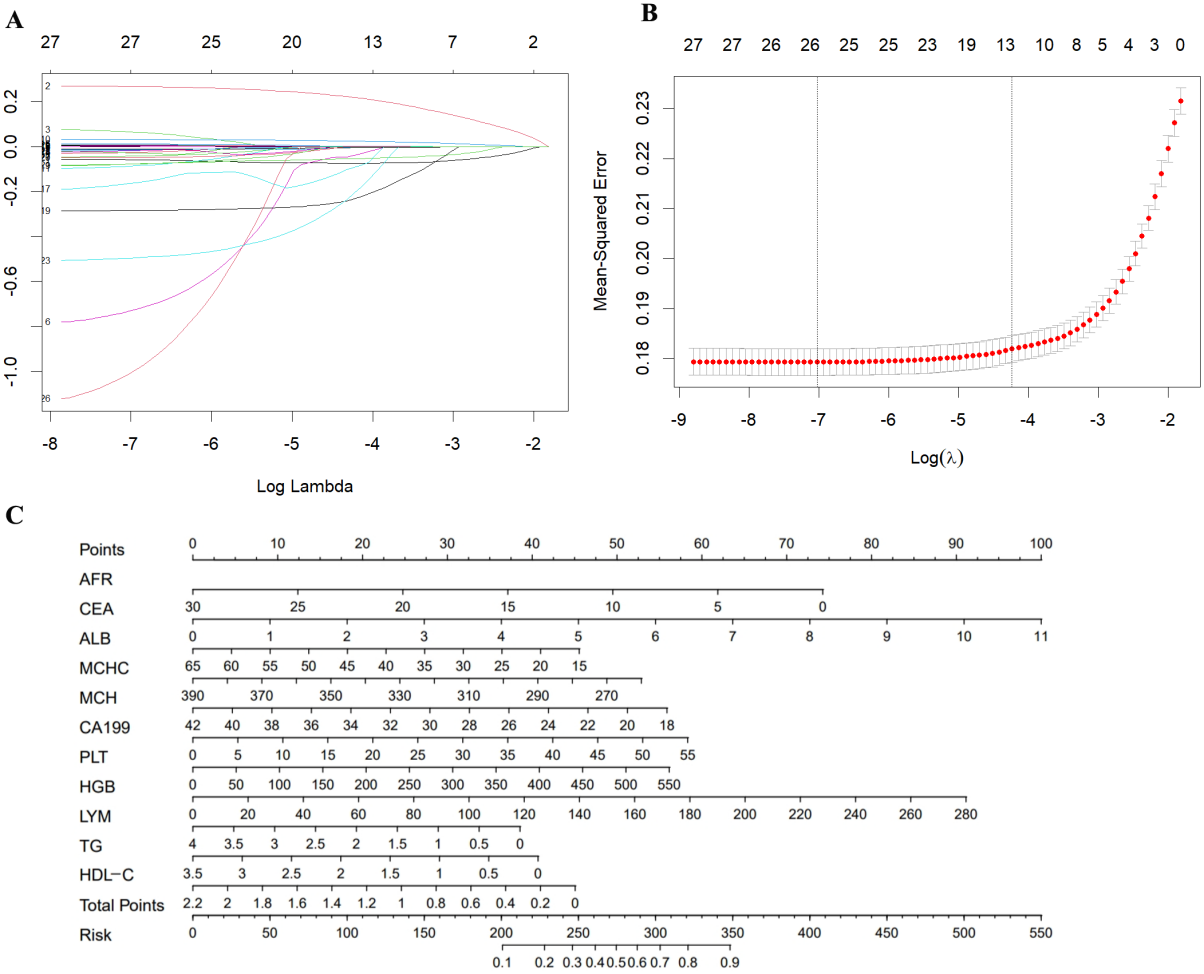
**(A)** A coefficient profile plot was produced against the log (λ) value of the 28 variables. **(B)** The partial likelihood deviance (binomial deviance) curve was plotted versus log (λ). Dotted vertical lines were drawn at the optimal values by using the minimum criteria and the 1 SE of the minimum criteria (the 1-SE criteria). **(C)** Nomogram model for the early diagnosis of GC. SE, standard error.

A univariate logistic regression analysis of the training set included the 29 laboratory indicators with statistically differences. The results showed that two of these indicators (RBC and WBC) had no statistically significant differences between the case group and the control group. The results of the univariate logistic regression analysis are shown in **Table 3**.To reduce model complexity, minimize multicollinearity among variables, prevent overfitting, and improve model generalization ability, this study conducted a LASSO regression analysis on the 27 variables that were statistically significant. **Figure 2A** illustrates the gradual shrinkage of original independent variable coefficients. Eventually, some coefficients were shrunk to zero, thereby avoiding model overfitting. The application of tenfold cross-validation technique, as shown in **Figure 2B**, selected the optimal number of variables within one standard error. Through LASSO regression analysis, this study identified 12 significantly correlated variables, namely CEA, CA199, LYM, HGB, MCH, MCHC, PLT, ALB, AGR, TG, HDL, and AFR.

**Table 3:** Univariate logistic regression analysis in the training set.

Variables	OR	95%CI	*P*
CEA	1.408	(1.350, 1.469)	< 0.001
CA125	1.040	(1.033, 1.047)	< 0.001
CA199	1.047	(1.040, 1.054)	< 0.001
WBC	1.024	(0.990, 1.059)	0.169
NEUT	1.071	(1.029, 1.114)	< 0.001
LYM	0.646	(0.572, 0.731)	< 0.001
MO	2.159	(1.411, 3.306)	< 0.001
RBC	0.928	(0.858, 1.004)	0.062
HGB	0.992	(0.989, 0.994)	< 0.001
MCV	0.954	(0.945, 0.964)	< 0.001
MCH	0.870	(0.850, 0.890)	< 0.001
MCHC	0.976	(0.972, 0.980)	< 0.001
RDW-SD	1.022	(1.010, 1.034)	< 0.001
RDW-CV	1.145	(1.105, 1.186)	< 0.001
PLT	1.004	(1.004, 1.005)	< 0.001
MPV	0.876	(0.831, 0.924)	< 0.001
PDW	0.943	(0.923, 0.964)	< 0.001
FIB	1.787	(1.643, 1.943)	< 0.001
TT	0.865	(0.821, 0.912)	< 0.001
ALB	0.917	(0.903, 0.930)	< 0.001
GLB	1.028	(1.012, 1.043)	< 0.001
AGR	0.230	(0.179, 0.297)	< 0.001
TC	0.821	(0.762, 0.885)	< 0.001
TG	0.621	(0.552, 0.698)	< 0.001
HDL	0.487	(0.374, 0.633)	< 0.001
NLR	1.134	(1.092, 1.178)	< 0.001
PLR	1.005	(1.004, 1.006)	< 0.001
LMR	0.825	(0.790, 0.861)	< 0.001
AFR	0.859	(0.843, 0.875)	< 0.001

### Development of a nomogram model for early diagnosis of GC

We conducted a multivariable logistic analysis on the aforementioned 12 variables (**Table 4**), revealing that 11 variables are independent diagnostic factors with statistical significance. These variables include CEA (odds ratio [OR] = 1.311, 95% CI = 1.251-1.373, P < 0.001), CA199 (OR = 1.032, 95% CI = 1.025-1.040, P < 0.001), LYM (OR = 0.751, 95% CI = 0.644-0.875, P < 0.001), HGB (OR = 1.010, 95% CI = 1.006-1.014, P < 0.001), MCH (OR = 0.933, 95% CI = 0.899-0.969, P < 0.001), MCHC (OR = 0.988, 95% CI = 0.981-0.995, P < 0.001), PLT (OR = 1.003, 95% CI = 1.002-1.004, P < 0.001), ALB (OR = 0.974, 95% CI = 0.952-0.996, P = 0.024), TG (OR = 0.706, 95% CI = 0.614-0.812, P < 0.001), HDL (OR = 0.541, 95% CI = 0.384-0.762, P < 0.001), and AFR (OR = 0.929, 95% CI = 0.907-0.953, P < 0.001). A diagnostic model for the training group was constructed based on these 11 independent variables, visualized using a nomogram (**Figure 2C**). Each variable’s values were assigned scores on the scale axis based on the magnitude of their regression coefficients. The sum of individual scores yielded a total score, and the probability of GC occurrence was calculated along the total score scale axis.

**Table 4:** Multivariate logistic regression analysis of the clinical parameters in the training set.

Variables	OR	95%CI	*P*
CEA	1.311	(1.251, 1.373)	< 0.001
CA199	1.032	(1.025, 1.040)	< 0.001
LYM	0.751	(0.644, 0.875)	< 0.001
HGB	1.010	(1.006, 1.014)	< 0.001
MCH	0.933	(0.899, 0.969)	< 0.001
MCHC	0.988	(0.981, 0.995)	< 0.001
PLT	1.003	(1.002, 1.004)	< 0.001
ALB	0.974	(0.952, 0.996)	0.024
AGR	0.943	(0.672, 1.324)	0.736
TG	0.706	(0.614, 0.812)	< 0.001
HDL	0.541	(0.384, 0.762)	< 0.001
AFR	0.929	(0.907, 0.953)	< 0.001

### Evaluation, analysis, and validation of the diagnostic nomogram model

**Figure 3:**
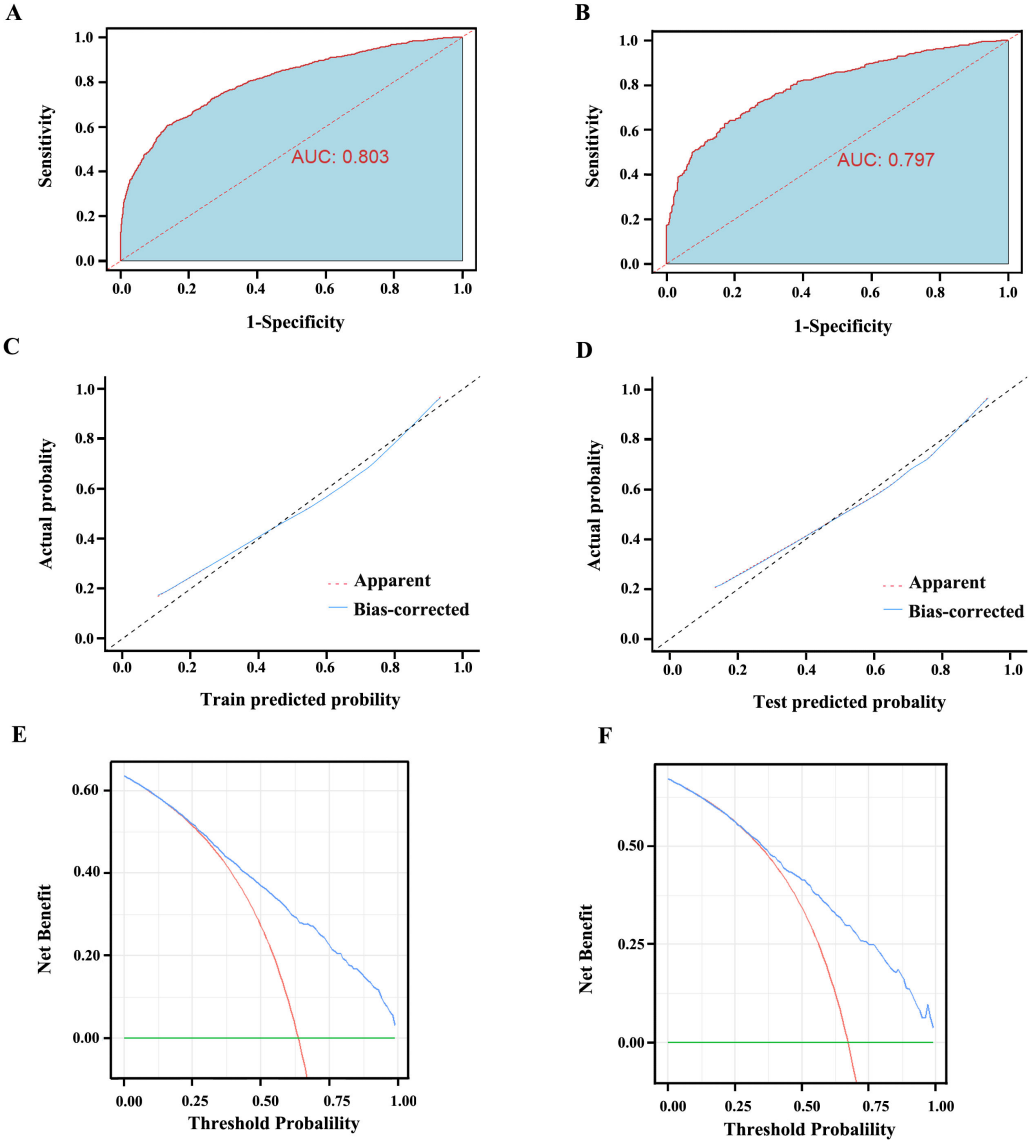
The evaluation curves of the Nomogram model. **(A)** AUC of the training set is 0.803 (95% CI: 0.787 ~ 0.818). **(B)** AUC of the validation set is 0.797 (95% CI: 0.772 ~ 0.821). **(C) **The calibration curve of the Nomogram model in the training set. **(D)** The calibration curve of the Nomogram model in the validation set. The diagonal line represents the reference line of complete coincidence between the predicted value and the actual value, The apparent red dotted line represents the actual situation of the Nomogram model, and the bias-corrected blue solid line represents the actual situation of the Nomogram model after correction. **(E)** The decision curve of the Nomogram model in the training set. **(F)** The decision curve of the Nomogram model in the validation set. The y-axis represents standardized net benefit. AUC, area under the receiver operating characteristic curves.

We initially plotted the ROC curve of the model in the training set (**Figure 3A**), with the AUC of 0.803 (95% CI: 0.787-0.818), sensitivity of 0.615, and specificity of 0.856, indicating good clinical diagnostic capability of the model. As the nomogram model was constructed based on the training set, we evaluated and validated it using a nomogram in the validation set, resulting in a slightly lower AUC of 0.797 (95% CI: 0.772-0.821) (**Figure 3B**). The calibration curves for the model were plotted in both the training set (**Figure 3C**) and validation set (**Figure 3D**), demonstrating good fitting and calibration capabilities of the model. Decision curve analysis in the training set (**Figure 3E**) and validation set (**Figure 3F**) revealed that the predictive model occupies a high position on the decision curve, indicating a higher net benefit and clinical utility. Furthermore, we compared the diagnostic efficacy for GC of each indicator alone and in combination within groups (**Table 5**). The AUC of the five subgroups, including tumor markers, infectious indicators, coagulation function indicators, lipid metabolism indicators and nutritional Index, were 0.720, 0.655, 0.705, 0.615 and 0.655, respectively, which were all lower than the AUC of the nomogram model.

**Table 5:** The diagnostic value of individual and combined indicators for gastric cancer.

Variables	AUC	95%CI	Sensitivity	Specificity
Tumor Biomarkers				
CEA	0.689	0.670-0.708	0.459	0.837
CA199	0.618	0.598-0.637	0.324	0.916
Combination	0.720	0.702-0.738	0.488	0.866
Infectivity Index				
LYM	0.576	0.555-0.598	0.734	0.394
HGB	0.577	0.556-0.598	0.481	0.656
MCH	0.627	0.607-0.647	0.396	0.828
MCHC	0.631	0.611-0.651	0.541	0.665
Combination	0.655	0.635-0.675	0.525	0.730
Coagulation Index				
PLT	0.600	0.580-0.621	0.541	0.617
AFR	0.701	0.682-0.721	0.668	0.659
Combination	0.705	0.686-0.724	0.696	0.624
Lipid Metabolism Index				
TG	0.568	0.546-0.590	0.704	0.435
HDL	0.560	0.538-0.582	0.714	0.415
Combination	0.615	0.594-0.636	0.702	0.489
Nutritional Index				
ALB	0.655	0.635-0.676	0.635	0.624

## Discussion

Gastric cancer imposes a substantial disease burden globally (1, 20). It is essential to identify high-risk populations early and detect GC to improve patient prognosis. Recently, an increasing number of studies have focused on the early prediction and diagnosis of GC, exploring and developing risk prediction methods and diagnostic models. The “ABC method” developed by MIKI et al. combines serum anti-Helicobacter pylori (Hp) IgG antibody and serum pepsinogen (PG) levels to identify individuals at high risk of developing GC in the future (21). The method (22) developed by Tu et al., which includes five biomarkers—serum G-17, PG I, PG II, PG I/II ratio, and anti-Hp IgG antibody—along with the afore mentioned “ABC method,” has shown limited predictive performance, with AUCs of less than 0.60. A multicenter cross-sectional study in China developed a GC risk prediction method incorporating seven variables (sex, age, G-17 level, PG I/II ratio, H. pylori infection, pickled food, and fried food), demonstrating good discriminatory ability with the AUC of 0.76 (23). Furthermore, most current studies tend to explore GC predictive factors from a micro perspective. The detection of indicators included in these models often requires significant time and may increase the financial burden on patients, affecting the clinical practicality of the models (24–26).

Currently, serum biomarkers are a minimally invasive, cost-effective, convenient, and repeatable tool for tumor diagnosis, which can detect disease progression relatively quickly. However, due to the insufficient sensitivity and specificity of a single biomarker for the diagnosis of GC, many studies often employ multiple biomarkers for combined detection to effectively enhance diagnostic efficacy. A nomogram integrates multiple predictive indicators to construct a multifactorial regression model, presenting the predicted probability of a clinical event as a score in a graphical format. This approach has been widely used to evaluate disease prognosis or predict disease diagnosis (27). In this study, we initially collected clinical data from 4283 patients who met the inclusion criteria and initially incorporated 36 variables, encompassing not only major laboratory test results but also the ratios of certain indicators. Through logistic regression and Lasso regression analysis, we finally identified 11 independent indicators associated with GC, including CEA, CA199, LYM, HGB, MCH, MCHC, PLT, ALB, TG, HDL, and AFR, and incorporated these indicators to develop a nomogram model for the diagnosis of GC.

Serum markers for tumor are widely used in the diagnosis, prognosis evaluation, and monitoring of tumors (28). CEA is a serum glycoprotein polymer primarily present in the human digestive system, playing an important role in regulating tumor cell proliferation and differentiation (29, 30). Elevated CEA levels are closely associated with tumor burden and are commonly used for predicting and diagnosing malignant tumors of the digestive tract (31). Additionally, CA19-9 levels are associated with tumor depth, lymph node metastasis (LNM), and tumor staging (32). Studies have shown that serum CA19-9 levels are significantly higher in GC patients with metastases. In our study, CEA and CA19-9 were identified as independent indicators associated with GC. The AUC for diagnosing GC was 0.618 for CEA and 0.619 for CA19-9, with a combined AUC of 0.720, indicating a higher diagnostic efficacy.

Inflammatory responses play a crucial role in various stages of tumor development, including growth, infiltration, invasion, and metastasis (33). Lymphocytes are a vital component of the body’s immune response and exert anti-tumor immune effects (34). Elevated lymphocyte counts have been associated with favorable prognoses in various cancers (35). In this study, LYM in GC patients were significantly lower than the control group. LYM was identified as an independent indicator of GC, with an AUC of 0.576 for diagnosing GC. Additionally, the inflammatory conditions may inhibit bone marrow hematopoietic function, potentially leading to reduced HGB levels (36). Our study found that the combined diagnostic AUC for GC of HGB (AUC=0.577), MCH(AUC=0.627), MCHC (AUC=0.631) and LYM was 0.655, which is higher than the diagnostic efficacy of each of these four indicators alone.

In the coagulation function indicators, PLT and AFR were included in the nomogram model of this study. During the development of malignant tumors, tumor cell infiltration, destruction, and metastasis can lead to a hypercoagulable state (37). Studies have shown that FIB levels of GC patients are associated with clinical stage, lymph node metastasis, and local infiltration depth (38). Our reach revealed that serum PLT levels in GC patients were notably higher than in those with benign gastric diseases, indicating a high risk of hypercoagulability in GC patients. The AUC for diagnosing GC was 0.600 for PLT and 0.701 for AFR, with a combined AUC of 0.705, indicating a higher diagnostic efficacy when both indicators are used together.

The development and progression of tumors are closely linked to lipid metabolism abnormalities (18). Studies have found that serum levels of LDL-C, HDL-C and TG in GC patients are higher than in normal individuals, whereas HDL-C levels are lower (39). However, our study indicates that serum TG levels in GC patients are lower than in patients with benign gastric diseases. This could be associated with inadequate consumption, increased tumor consumption, and a continuous decline in nutritional status as GC progresses. We identified TG (AUC=0.568) and HDL (AUC=0.560) as independent indicators associated with GC, with a combined AUC of 0.615 for diagnosing GC. Additionally, in this study, serum albumin (ALB) levels, which reflect the nutritional status of the body, were significantly lower in GC patients than those with benign gastric diseases. The AUC for diagnosing GC using ALB alone was 0.655.

Our nomogram model showed high diagnostic performance for GC in both the training set (AUC=0.803) and validation set (AUC=0.797), which was significantly higher than the diagnostic efficacy of individual or combined indicators included in the model. An article published in JAMA in 2017 emphasized that calibration or goodness of fit is often considered the most important feature of predictive models, as it reflects the model’s ability to accurately estimate absolute risk (40). In this study, calibration curves were plotted for the model in both the training and validation sets, showing good model fit and calibration ability, which indicates excellent performance. The clinical utility of predictive models has also been widely discussed as an important feature for evaluating models in recent years. This evaluation is primarily based on decision curve analysis to assess whether the model can benefit patients by influencing clinical decisions (41). The clinical utility of predictive models is mainly evaluated through decision curve analysis, and we also plotted decision curves for both sets, demonstrating substantial net benefit produced by the model. The main strengths of this study lie in its large sample size and the inclusion of key laboratory indicators. The variables included in the model are generally obtainable in most hospitals, and test results can be obtained within 24 hours of admission, ensuring the practical application of the diagnostic model in clinical settings.

However, this study also has some limitations. The study was retrospective and could introduce bias that may affect the causality and interpretation of the results. Some cases and other potentially meaningful indicators, such as PG I, PG II, PG I/II, CA724, NSE, D-dimer, CK-MB, CK, LDH, and HCY were excluded due to data loss. While the absence of these indicators may underestimate the predictive power of the model, inclusion of these indicators and filling in the missing data may affect the authenticity of the data, thereby affecting the predictive power of the model. Additionally, this study is single-center, and both the training and validation datasets are from one hospital, with only internal validation conducted, lacking external validation. Therefore, based on the existing results, our next step is to conduct a multi-center prospective study, gathering comprehensive clinical data of patients, to further improve the early diagnosis of GC.

## Conclusion

Based on the analysis of large sample size, we constructed a nomogram model with 11 routine laboratory indicators, which showed good diagnostic efficacy and calibration, providing a convenient visualization tool and new possibilities for the early diagnosis of GC.

## Data Availability

The raw data supporting the conclusions of this article will be made available by the authors, without undue reservation.
